# Environmental Impacts from Pesticide Use: A Case Study of Soil Fumigation in Florida Tomato Production

**DOI:** 10.3390/ijerph8124649

**Published:** 2011-12-14

**Authors:** Doris Sande, Jeffrey Mullen, Michael Wetzstein, Jack Houston

**Affiliations:** Department of Agriculture and Applied Economics, Conner Hall, The University of Georgia, Athens, GA 30602, USA; Email: dwangila@uga.com (D.S.); mwetz@uga.edu (M.W.); jhouston@uga.edu (J.H.)

**Keywords:** pesticide use, environmental impacts, ozone depletion potential, Environmental Impact Quotient (EIQ), field use rating, fumigants, alternatives, methyl bromide

## Abstract

The search for alternative fumigants has been ongoing since the 1992 Parties of the Montreal Protocol classified methyl bromide as a Class I controlled substance with an ozone depletion potential (ODP) of 0.7 and destined it for phase-out. This paper focuses on the hazards from fumigants proposed as alternatives for pre-plant soil fumigation in tomato production. We use the Environmental Impact Quotient (EIQ) developed by Kovach *et al.* to estimate the hazards from methyl bromide and the proposed alternative fumigants to workers, consumers, beneficial arthropods, birds, fish, and bees. Our findings indicate that iodomethane 98/2 has the lowest EIQ index value and field use rating, and is the alternative with the lowest relative risk. Among environmental categories, workers and beneficial arthropods experience the highest relative risks from the proposed tomato fumigants, and fish and consumers the least risks.

## 1. Introduction

Methyl bromide (MeBr) is a broad spectrum agricultural pesticide that has been used on more than 100 crops worldwide—mainly in strawberry, bell pepper and tomato production [1]. Its primary use is as a soil fumigant to control soil-borne diseases, nematodes, soil insects, pathogens and weeds. It is also used in post-harvest storage and transportation of mainly fruits and vegetables; for laboratory and analytical use as a feedstock; and for quarantine and pre-shipment (QPS) purposes. 

Despite widespread use of methyl bromide, this fumigant has been found to cause stratospheric ozone layer depletion and to be associated with serious health effects. Methyl bromide has also been associated with effects to soil biodiversity and groundwater contamination [2]. In response to these findings, the 1992 Parties of the Montreal Protocol labeled it an ozone depleting substance (ODS) with an ozone depletion potential of 0.7 [3,4]. The U.S. Environmental Protection Agency (US EPA) placed it under the U.S. Clean Air Act of 1990 for regulation. Under the 1998 amendment to this Act, the importation and production of methyl bromide was to be reduced by 50% by the year 2001, 70% by 2003, and total phase-out by 2005 [5,6,7,8]. To date, exemptions to the phase-out have been allowed for quarantine and pre-shipment (QPS), critical use, and chemical feedstock uses. Nonetheless, there has been a heightened search for alternative pesticides to serve as substitutes to methyl bromide across its various uses. 

The Methyl Bromide Technical Options Committee [5] defined ‘alternatives’ as “any practice or treatment that can be used in place of methyl bromide. ‘Existing alternatives’ are those alternatives in present or past use in some regions. ‘Potential alternatives’ are those in the process of investigation or development”. The search for alternatives has been hampered by findings indicating that risks from such substitute chemicals may be as great as those from the pesticides being banned [9], and by the challenges due to regional differences in soil types and weather conditions that may lead to discrepancies in the efficacy of the alternatives [10]. While research on methyl bromide alternatives is ongoing, the US Environmental Protection Agency (EPA) continues to prioritize the registration of alternatives for various agricultural sectors. 

Pesticides, by their very nature, are designed to impede and/or prevent the development of living organisms, to interfere with their ability to reproduce, or to kill them outright. While their use is generally undertaken to target specific organisms, pesticide applications often cause harm to non-target species. Due to differences in chemical composition, mode of action, and application techniques, the substitution of one pesticide for another may result in different effects on non-target populations. The focus of this paper is to derive a measure by which to compare the relative, potential impact of alternatives to methyl bromide on non-target populations. These include relative impacts to workers, consumers, birds, bees, fish, and beneficial arthropods. The measure we derive is based on the chemical composition of the pesticides and their expected application rates, not on regulatory guidelines or restrictions related to the handling or application of the pesticides. We limit our analysis to the alternative proposed for tomato production in Florida, which are shown in Table 1. 

**Table 1:** Primary methyl bromide alternatives for the Florida Tomato Sector.

Alternatives available	Alternatives under development
1,3-Dichloropropene (1,3-D)	Dimethyl Disulfide
Chloropicrin	Furfural
Dazomet	
Iodomethane	
Metam Sodium	
Glyphosate (H)	
Paraquat (H)	
Halosulfuron-methyl (H)	
s-Metolachlor (H)	
Trifloxysulfuron-methyl (H)	
Rimsulfuron (H)	
Fosthiazate	

Source: US EPA—Methyl Bromide Alternatives [11].

Historically, the United States (US) has consumed the bulk of total methyl bromide annually, 42 million pounds (29.4 percent) of the World’s 143 million pounds [12]. Of this share, 83 percent was used for pre-plant soil fumigation, 11 percent for post-harvest treatment of stored commodities, and 6 percent for fumigation of structures [1]. The National Center for Food and Agricultural Policy (NCFAP) estimated that about 35 million pounds (active ingredient) of methyl bromide were used annually for pre-plant soil fumigation in the US [13].

Florida is one of the two leading methyl bromide users in the US because its weather conditions favor higher pest populations. In 1997, Florida accounted for 36 percent of the US pre-plant methyl bromide use [1]. According to a 1996 survey, Florida crops depending on methyl bromide for fumigation included tomato (53 percent), bell peppers (33 percent), strawberry (12 percent), and eggplant (2 percent) [14]. At that time, methyl bromide was applied in combination with chloropicrin to more than 80 percent of Florida’s tomato acreage [15]. The most commonly used formulation of methyl bromide in Florida has historically been the 67/33, which has 67 percent methyl bromide and 33 percent chloropicrin. 

Various studies have demonstrated that there is no perfect one-to-one replacement for all of the uses of methyl bromide [2,4]. The main challenges are in relation to efficacy (due to the broad spectrum activity), cost, ease of use, availability, and worker and environmental safety. One recommendation is to combine the activity of various active ingredients and improve fumigant retention in the soil using mulches [16]. Most of the alternatives registered so far are effective either against nematodes, diseases, or both, but with little to no weed management activity. It is therefore recommended to mix the fumigants with herbicides and insecticides, a measure that greatly increases their cost. The proposed herbicides include glyphosate, paraquat, halosufuron methyl, s-metachlor, rimsulfuron, and trifloxysulfuron methyl [11]. A qualitative measure of the effectiveness of the soil fumigant alternatives is shown in Table 2.

**Table 2:** Florida maximum use rates and effectiveness of soil fumigant alternatives.

Fumigant chemical	Max. use rate	Relative pesticidal activity		
		Nematode	Disease	Weed
Methyl bromide 67/33	350 lb	Excellent	Excellent	Good–Excellent
Chloropicrin	300 lb	None–Poor	Excellent	Poor
Methyl iodide	350 lb	Good–Excellent	Good–Excellent	Good–Excellent
Metam sodium (Vapam)	75 gal	Erratic	Erratic	Erratic
Telone II	18 gal	Good–Excellent	None–Poor	Poor
Telone C17	26 gal	Good–Excellent	Good	Poor
Telone C35	35 gal	Good–Excellent	Good–Excellent	Poor
Pic-Clor 60	250 lb	Good–Excellent	Good–Excellent	Poor
Metam Potassuim (Kpam)	60 gal	Erratic	Erratic	Erratic

The United States Department of Agriculture’s (USDA) National Agricultural Pesticide Impact Assessment Program (NAPIAP) carried out one of the first economic impact assessments of the phase-out of methyl bromide [17]. They concluded that the phase out of MeBr as a fumigant would result in a substantial impact on many commodities because available alternatives are either less effective or more expensive than MeBr [17,18], and that the effect would be most felt in Florida and California. They also estimated that the effects to fumigation would be $1.5 billion dollars in annual lost production in the United States alone. This estimate does not include post-harvest, non-quarantine uses and quarantine treatments of imports and other future economic aspects such as lost jobs, and markets. The report predicted the major crop losses would occur with tomatoes ($350 M), ornamentals ($170 M), tobacco ($130 M), peppers ($130 M), strawberries ($110 M) and forest seedlings ($35 M). 

The nature and extent of pesticide impacts on the environment, non-target species, and human beings, vary to a great degree depending on their inherent chemical properties and the manner in which these chemicals are incorporated into the environment. The environmental behavior (mobility and persistence) and toxicity profiles for most pesticides differ from each other too. Merely reducing the amount of pesticides applied to crops may not provide sufficient insight into their environmental impacts [19]. Thus, even though pesticide risk to the environment is related to the amount of active ingredient applied, total pounds of active ingredient applied per year is not the best indicator of risk [20]. The interplay among these factors, together with the degree of exposure of organisms to the chemicals dictates the degree of pesticide impacts. In addition, climatic conditions, soil properties, topography, and many other site-specific factors influence pesticide behavior, consequently affecting risk and hazard levels [21].

Pesticide risk is often defined as the inherent potential to cause harm coupled with the likelihood of exposure. Numerous pesticide risk indicator models exist for the calculation of environmental pesticide risk. These models differ in four main aspects: (1) the components of the analysis, including pesticides considered, variables assessed, and the choice of specific measurable endpoints as the indicators of impacts on these variables; (2) the mathematical structure of the model (e.g., relative weighting of variables and scoring of the results); (3) the method for filling data gaps; and 4) whether usage data were factored into the analysis [22]. 

Several methods have been used to assign an economic value to pesticide impacts. Such methods include: remediation cost, lost productivity, and willingness-to-pay (WTP) to avoid pesticide risk. WTP, which is commonly used, does not measure the existence or extent of an environmental problem, rather it measures the attitude toward a problem, and whether the problem bothers a particular stakeholder enough to pay for an alternative [23]. 

Environmental impacts of pesticide use were commonly proxied through variables such as pounds of active ingredient (A.I.) applied or dollars spent on pesticides [24]. The disadvantage is that both these measures assume environmental damage is directly correlated with the quantity of pesticide used, regardless of the specific chemical formulation [24]. The increased availability of low-dosage alternatives lend credence to the argument that weight and volume measures are not adequate proxies for assessing pesticide risk [25].

Cornell University’s Environmental Risk Analysis Program has identified eight of the indicators widely used worldwide: Environmental Potential Risk Indicator for Pesticides (EPRIP), Environmental Yardstick for Pesticides (EYP), Survey of National Pesticide Risk Indicators (SYNOPS), System for Predicting the Environmental Impact of Pesticides (SyPEP), Pesticide Environmental Risk Indicator (PERI), Environmental Impact Quotient (EIQ), Chemical Hazard Evaluation for Management Strategies (CHEMS1), and Multi-Attribute Toxicity Factor (MATF). The first four indicators are referred to as predicted environmental concentration (PEC) indicators, and the later four constitute ranking indicators. 

In this paper we concentrate on the Environmental Impact Quotient (EIQ), an indicator which expresses the relative impact of pesticides on the environment by scoring their effects on a set of environmental categories [26]. The EIQ is a model that transforms the environmental impact information related to pesticide active ingredients to a single value. Lower values of the EIQ indicate lower risk of negative environmental impacts.

In the following section, we present the data and methodology used in our study. Section 3 presents a discussion of our findings, and conclusions follow in Section 4.

## 2. Methods

In our study, we used environmental categories similar to Mullen *et al.* [20], dividing the environmental pesticide impacts into eight categories. These categories include acute human health, chronic human health, aquatic species, birds, other mammals, arthropods, groundwater, and surface water. This categorization accounts for the problem that a given pesticide is likely to pose different levels of risk to different environmental components. The EIQ index in our study accounts for these categories through equation 1. Table 3 provides definitions of the variables in equation 1 and the criteria for assigning values to those variables. 


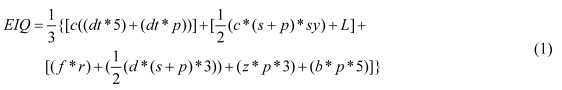


Table 4 presents the values assigned to each variable across the set of proposed alternatives to methyl bromide in Florida tomato production. These values were obtained from different sources including International Union of Pure and Applied Chemistry 250 (IUPAC), Extension Toxicology Network (EXTOXNET), and the Crop Data Management System (CDMS) 251 which provides labels and material safety data sheets (MSDS). The list of alternatives to methyl bromide for use on Florida tomatoes was obtained from the US Environmental Protection Agency (US EPA).

**Table 3:** Definition for symbols and ratings for each toxicity category in Equation (1).

Environmental category	Symbol	Score 1	Score 3	Score 5
Long-term health effects (chronic)	c	Little-none	Possible	Definite
Dermal toxicity (Rat LD50)	dt	>2000 mg/kg	200–2000 mg/kg	0–200 mg/kg
Bird toxicity (8 day LC50)	d	>1000 ppm	100–1000 ppm	1–100 ppm
Bee toxicity	z	Non-toxic	Moderately toxic	Highly toxic
Beneficial. Arthr. Toxicity.	b	Low impact	Moderate	Severe impact
Fish toxicity (96 h LC50)	f	>10 ppm	1–10 ppm	<1 ppm
Plant surface half-life	p	1–2 weeks (pre-emerg. Herbic.)	2–4 weeks (post-emerg. herbic.	>4 weeks
Soil residue half-life (TI/2)	s	<30 days	30–100 days	>100 days
Mode of Action	sy	Non-system; all herbicides	Systemic	
Leaching potential	L	Small	Medium	Large
Surface runoff potential	r	Small	Medium	Large

Source: Kovach *et al.* [26].

**Table 4:** Methyl bromide and alternative fumigant risk levels.

Active ingredient	Chronic health	Dermal toxicity	Plant surface ½ life	Soil residue ½ life	Mode of action	Leaching potential	Fish toxicity	Surface potential	Bird toxicity	Bee toxicity	Ben. Arthropod toxicity
**Fumigants**											
MeBr	5	5	-	1	1	1	3	1	3	1	1
1,3-D (*Telone*)	3	3	-	1	1	3	3	1	3	3	3
Chloropicrin	1	5	-	1	1	1	5	1	5	3	1
Iodomethane (*Midas*)	3	1	1	1	1	1	1	1	3	-	3
Metam sodium	5	5	-	1	1	3	5	5	1	3	1
**Herbicides**											
Glyphosate	1	5	1	1	1	1	3	5	1	3	3
Paraquat	5	3	5	5	1	1	3	5	3	1	1
s-Metachlor	3	1	1	3	1	3	3	3	1	5	1
Trifloxysulfuronmethyl	0	1	3	1	1	3	1	5	1	1	1

Source: Risk levels from [26], EXTOXNET, IUPAC, Mullen [20].

The EIQ formula can be broken down into effects on the farm worker (applicator and harvestor), consumer (exposure and groundwater effects), and ecology (fish, birds, bees, other beneficial insects), as shown in Table 5. 

**Table 5:** EIQ equation environmental components.

EIQ equation component	Equation
Farm worker (applicator + harvester)	c*((dt*5) + (dt*p))
Consumer (exposure + groundwater effects)	(c*(s + p)/2*sy) + (L)
Ecology (fish, birds, bees, other beneficial insects)	(f*r) + (d*(s+p)/2*3) + (z*p*3) + (b*p*5)
Total EIQ = farm worker + consumer + ecology	{[c*(dt*5)+(dt*p)]+[(c*(s+p)/2*sy)+(L)+[(f*r)+(d*(s+p)/2*3)+(z*p*3)+(b*p*5)]}/3
Field Use EIQ	EIQ * % active ingredient * rate/acre

The EIQ criterion has been used widely to assess the relative risk associated with adopting various pesticides or pest management programs, including integrated pest management (IPM) programs. [26]. Direct comparison between pesticides is most informative when they can be used to control the same pest. Methyl bromide alternatives are essentially supposed to control the same pests as methyl bromide.

In addition to the standard EIQ, we derive a Field Use EIQ that accounts for different formulations of the same active ingredients. While the EIQ is specific to individual active ingredients, the Field Use EIQ is specific to individual pesticide formulations, which may have multiple active ingredients. Additionally, the Field Use EIQ is weighted by the pesticide’s application rate. As shown in equation 2, the Field Use EIQ for pesticide formulation (p) is the sum of the EIQ’s of the individual active ingredients (a) weighted by both the proportion of the formulation each active ingredient comprises and the application rate (lb/acre) of the formulation. As with the standard EIQ, higher values of the Field Use EIQ indicate greater relative risk. 

Field Use EIQ_p_ = Σ_a_(EIQ_a_ × % active ingredien_a_) × Rate_p_                                        (2)

where the subscript p refers to the pesticide formulation, and the subscript a refers to individual active ingredients.

Most of the proposed alternative fumigants are combinations of two or more chemicals. The two primary fumigant formulations for Florida tomatoes are combinations of 1,3-dichloropropene (Telone), and iodomethane (Midas). It is often recommended to include herbicides with the alternatives due to their poor weed suppression. To account for this, Field Use EIQs are also derived for the fumigants plus various combinations of pre- and post-emergence herbicides for maximum weed control, as recommended by Noling *et al.* [27]. 

## 3. Results

Field Use EIQ ratings generated for methyl bromide and various formulations of the recommended alternative fumigants are reported in Table 6. Midas products have broad spectrum activity, so they were evaluated without a chloropicrin follow-up application. Telone products, on the other hand, have little disease activity, and no weed control activity. They were thus evaluated with a sequential application of chloropicrin and various recommended herbicides.

The main herbicides recommended are a mixture of napropamide and s-metolachlor as pre-emergent herbicides, followed by one or two applications of halosulfuran. For comparison purposes, we evaluated glyphosate (pre-emergent) and three other post-emergent herbicides: paraquat, trifloxysufuron methyl, and rimsulfuron. 

**Table 6:** Fumigant and herbicide characteristics.

Product	Formulation	Application rate (lb/acre)	EIQ	Field use EIQ
**Fumigants**				
	% Methyl Bromide/% Chloropicrin			
Methyl bromide	67/33	200	53.6 [26]	10,720
	% Iodomethane/% Chloropicrin			
Midas 98:2	97.8/1.99	137.5	16.2 [c]	2,223
Midas EC Bronze	49.9/44.78	275	26.8 [c]	7,273
Midas 50:50	49.9/49.75	275	29.0 [c]	7,951
Midas EC Gold	32.93/61.69	415	31.3 [c]	12,996
Midas 33:67	32.93/66.67	415	33.6 [c]	13,873
Midas 25:75	24.95/74.63	550	35.7 [c]	19,554
	% 1,3-Dichloropropene/% Chloropicrin			
Telone II (1,3-D)	97.5/0	121.2	27.8 [26]	3,285
Telone C17	81.2/16.5	147.9	29.6 [c]	4,372
Telone C35	63.4/34.7	187.6	32.3 [c]	6,067
Pic Clor 60	39/59.6	242	36.1 [c]	8,739
	% active ingredient			
Chloropicrin	96	150	42.4 [26]	6,106
Vapam (metam sodium)	42	320	26.6 [26]	3,575
**Herbicides**				
Glyphosate	41	2	15.33 [26]	12.57
Napropamide	24.1	2	12.57 [26]	6.06
s-metolachlor	83.7	0.95	22 [26]	17.49
Paraquat (post-)	30.1	0.54	92 [c]	14.95
Halosulfuron methyl	75	0.024	20.2 [26]	0.36
Rimsulfuron	25	0.125	15.84 [26]	0.5
Trifloxysulfuron methyl	75	0.006	12.67 [c]	0.06

Source: EIQ values from [26] and personal calculations [c] using values from IUPAC, CDMS, EXTOXNET, and MSDS.

From Table 6, methyl bromide has the highest EIQ value among all the fumigants in the study. However, the EIQs for both the Midas and the Telone products increase as the concentration of chloropicrin increases. 

The rank order of the fumigants changes when application rate is incorporated into the Field Use EIQ. With the exception of Midas 98:2, the Midas products all have higher application rates than methyl bromide, and higher concentrations of chloropicrin. Together, these factors increase the Field Use EIQ, so much so that half of the Midas products have a higher Field Use EIQ than methyl bromide. 

The Field Use EIQ of the Telone products also increases as application rate and the concentration of chloropicrin increase. Although they approach it, none of these products exceed the Field Use EIQ of methyl bromide.

Compared to the fumigants, the herbicides have similar EIQs. Their Field Use EIQs, however, are much lower than those of the fumigants. This is due to the lower application rates. 

Among the herbicides, paraquat has the highest EIQ (92), compared to values between 12 and 22 for the rest of the herbicides. The rank order of the herbicides changes considerably, however, under the Field Use EIQ. Although the active ingredient in paraquat has a high EIQ, the concentration of active ingredient is low relative to several of the other herbicides. Similarly, the application rate of paraquat is lower. With these factors considered, paraquat has the third highest Field Use EIQ.

Results of the Field Use EIQs for various formulations of Midas, Telone + chloropicrin, and Telone + Vapam, with and without herbicides are shown in Table 7. Because of the difference in magnitude of the Field Use EIQ between the fumigants and the herbicides, the addition of an herbicide treatment has little effect on the overall Field Use EIQ. However, these pesticides have different impacts on the environmental and human health components that comprise both the EIQ and the Field Use EIQ. 

**Table 7:** Field Use EIQ ratings of fumigants with and without herbicides (100 s).

Product	W/o herbicides	With pre-emergent herbicides		With nap + met and post-emergent herbicides				With glyphosate and post-emergent herbicides			
		glyp	nap + met	paraq	Halos	trif	rim	paraq	halos	glyp + trif	glyp + rim
Midas@ 98:2	22.2	22.4	22.5	22.6	22.5	22.5	22.5	22.5	22.4	22.2	22.4
Midas@ EC Bronze	73.7	73.8	74	74.1	74	74	74	74	73.8	73.7	73.8
Midas@ 50:50	79.5	79.6	79.7	79.9	79.7	79.7	79.8	79.8	79.6	79.5	79.6
Midas@ Gold	130	130.1	130.2	130.3	130.2	130.2	130.2	130.2	130.1	130	130.1
Midas@ 33:67	138.7	138.9	139	139.1	139	139	139	139	138.9	138.7	138.9
Midas@ 25:75	195.5	195.7	195.8	195.9	195.8	195.8	195.8	195.8	195.7	195.5	195.7
Telone II +Pic	93.9	94	94.1	94.3	94.1	94.1	94.2	94.2	94	93.9	94
Telone C17 + Pic	104.8	104.9	105	105.2	105	105	105	105.1	104.9	104.8	104.9
Telone C35 + Pic	121.7	121.9	122	122.1	122	122	122	122	121.9	121.7	121.9
Pic Clor 60 + Pic	148.5	148.6	148.7	148.8	148.7	148.7	148.7	148.7	148.6	148.5	148.6
Telone II +Vapam	68.6	-	-	-	-	-	-	-	-	-	-
Telone C17 Vapam	79.5	-	-	-	-	-	-	-	-	-	-
Telone C35 +Vapam	96.4	-	-	-	-	-	-	-	-	-	-
Pic Clor 60+ Vapam	123.1	-	-	-	-	-	-	-	-	-	-
MeBr	107.2	-	-	-	-	-	-	-	-	-	-

glyp = glyphosate, halos = halosulfuron methyl, met = s-metolachlor, nap = napropamide, paraq = paraquat, rim = rimsulfuron, trif = trifloxysulfuron methyl. Note: Methyl bromide and the treatments with Vapam do not need an additional herbicide application.

### 3.1. Impacts by Environmental Category

Table 8 presents the Field Use EIQ for each pesticide, broken down by environmental category. With the exception of Midas 25/75, all alternative fumigants have the highest impacts on workers, and second highest effects to beneficial arthropods. Methyl bromide, too, has the highest effects on workers and the second highest effects on birds. In contrast, the herbicides tend to pose the highest relative risk to beneficial arthropods, followed by workers. As one would expect, the highest impacts are among categories that come into direct contact with the products, namely, workers and beneficial arthropods.

**Table 8:** Field Use EIQ by environmental category.

Fumigant	Worker	Consumer + L	Fish	Birds	Bee	Beneficials
Midas@ 98:2	2,515	558	148	1,281	51	2,115
Midas@ EC Bronze	6,719	1,466	753	4,404	2,316	6,469
Midas@ 50:50	7,190	1,568	821	4,755	2,573	6,959
Midas@ EC Gold	11,292	2,454	1,417	7,817	4,816	11,220
Midas@ 33:67	12,005	2,608	1,520	8,349	5,204	11,961
Midas@ 25:75	16,631	3,607	2,190	11,796	7,721	16,761
Telone II + Pic	9,860	2,007	1,075	4,219	4,730	6,281
Telone C17 + Pic	10,781	2,203	1,202	4,855	5,221	7,173
Telone C35 + Pic	12,138	2,498	1,402	5,897	5,967	8,620
Pic Clor 60 + Pic	13,851	2,893	1,724	7,827	7,036	11,221
Telone II + Vapam	8,138	2,020	1,565	1,099	2,787	4,953
Telone C17 +Vapam	9,059	2,216	1,692	1,735	3,278	5,845
Telone C35 +Vapam	10,416	2,511	1,892	2,777	4,024	7,292
Pic Clor 60 +Vapam	12,129	2,906	2,214	4,707	5,093	9,893
MeBr	12,193	1,885	464	7,125	2,206	6,063
glyphosate (H)	6.56	2.46	4.1	4.92	7.38	12.3
paraquat (H)	24.38	4.23	2.44	7.31	2.44	4.06
halosulfuron methyl (H)	0.22	0.11	0.05	0.11	0.16	0.44
s-metachlor (H)	9.54	7.16	7.16	9.54	7.16	11.93
trifloxysulfuron methyl (H)	0	0.01	0.02	0.03	0.04	0.07
rimsulfuron (H)	0.25	0.09	0.03	0.19	0.32	0.6

In addition to the fact that adding chloropicrin (Pic) to Telone products results in higher environmental risks than using metam sodium (Vapam), the ordering of the impacts is also altered for some environmental categories. This is due to the fact that chloropicrin and Vapam have different effects on each of the environmental categories. Among all the alternatives, however, Midas 98/2 has the lowest Field Use EIQ across all the environmental categories, while Midas 25/75 has the highest. 

## 5. Conclusions

Increased awareness of environmental and human health effects from pesticide use in agriculture have prompted policy measures aimed at reducing these effects. One such policy measure is the decision to phase-out methyl bromide after the 1991 Montreal Protocol declared it as a Class I controlled substance. US chemical manufacturing companies, together with the Environmental Protection Agency, have actively been seeking alternative fumigants to methyl bromide for various agricultural sectors. Finding alternative fumigants to methyl bromide is especially important to the tomato industry. So far, it has been challenging to find broad spectrum alternatives, so several pesticides are combined to achieve such action. 

Using the EIQ and Field Use EIQ, our findings indicate that workers and beneficial arthropods are the most likely to be affected by all fumigants. This result is expected since workers are exposed to fumigants in their most concentrated form during mixing and application, while beneficials are exposed via their habitat and feeding habits. On the other hand, fish and consumers face lower relative risk from the tomato fumigants. The results also indicate that while chloropicrin is needed as a warning agent to all the alternatives, increasing the chloropicrin component in the fumigant formulation greatly increases its relative risk. It appears prudent to limit chloropicrin use to the minimal amounts necessary for efficacy. 

The impetus behind the methyl bromide phase-out is its ozone depleting potential. It is important to note that the EIQ and Field Use EIQ derived in this paper account for a limited scope of potential environmental impacts. For example, ozone depletion risk is not a component of either measure. Furthermore, regulatory guidelines or restrictions related to the handling or application of the pesticides are not accounted for in either measure. Nonetheless, The EIQ and Field Use EIQ are can help policy makers in evaluating the relative risks of proposed alternatives.

There is a wide array of environmental risk indicators for pesticides. Different indicators are developed for different purposes, and therefore may lead to very different conclusions. Indicators employing ranking methodologies are easier to use compared to predicted environmental concentration (PEC) indicators that are more data intensive. However, PEC indicators have been found to give a more complete picture of environmental risk 

Furthermore, there is no scientifically valid way of combining risks across various environmental and health categories. Indices use weighting schemes to reflect the relative importance of one category to another. Assigning relative weights is ultimately a subjective, political process. 

Lastly, indicators are relative measures, not direct measures of real risk. For this reason, they should be used as signposts only and should not be the sole basis for decision-making. These issues notwithstanding, relative risk indicators can facilitate regulatory and production decisions about which pesticides are used, and how they are used.
